# Sexual Dimorphism in the Musculoskeletal System: Sex Hormones and Beyond

**DOI:** 10.1210/jendso/bvae153

**Published:** 2024-09-01

**Authors:** Lilian I Plotkin, Angela Bruzzaniti, Roquelina Pianeta

**Affiliations:** Department of Anatomy, Cell Biology & Physiology, Indiana University School of Medicine, Indianapolis, IN 46202-5120, USA; Indiana Center for Musculoskeletal Health, Indiana University School of Medicine, Indianapolis, IN 46202-5120, USA; Richard L. Roudebush Veterans Administration Medical Center, Indianapolis, IN, USA; Indiana Center for Musculoskeletal Health, Indiana University School of Medicine, Indianapolis, IN 46202-5120, USA; Department of Biomedical and Applied Sciences, Indiana University School of Dentistry, Indianapolis, IN 46202-5120, USA; Department of Anatomy, Cell Biology & Physiology, Indiana University School of Medicine, Indianapolis, IN 46202-5120, USA; Indiana Center for Musculoskeletal Health, Indiana University School of Medicine, Indianapolis, IN 46202-5120, USA; Richard L. Roudebush Veterans Administration Medical Center, Indianapolis, IN, USA

**Keywords:** bone, skeletal muscle, sexual dimorphism, androgens, estrogens

## Abstract

Mounting evidence indicates that whereas some fundamental aspects of bone cell differentiation and function are similar in females and males, there is a clear contribution of sex/gender on the effects of signaling molecules on bone mass and strength and, consequently, on the effects of pharmacologic approaches to treat skeletal disorders. However, until recently, most studies were designed and performed using only 1 sex, resulting in a scarcity of published information on sexual dimorphism of the musculoskeletal system, including the mandible/masticatory muscles and the axial and appendicular bones and skeletal muscles. Further, it is now recognized that scientific rigor requires the study of both males and females. Therefore, there is an increasing need to understand the molecular and cellular basis for the differential outcomes of genetic manipulations and therapeutic agent administration depending on the sex of the experimental animals. Studies have shown higher muscle mass, cancellous bone mass, and long bone width in males compared with females as well as different traits in the pelvis and the skull, which are usually used for gender identification in forensic anthropology. Yet, most reports focus on the role of sex hormones, in particular, the consequences of estrogen deficiency with menopause in humans and in ovariectomized animal models. In addition, emerging data is starting to unveil the effects of gender-affirming hormonal therapy on the musculoskeletal system. We summarize here the current knowledge on the sex/gender-dependent phenotypic characteristics of the bone and skeletal muscles in humans and rodents, highlighting studies in which side by side comparisons were made.

Both human and murine studies have provided invaluable information for our understanding of bone and muscle physiology [1]. The phenomenon of sexual divergence in these tissues during the ontogenic process [2-6] is evidenced in the growth in length and thickness of long bones of the epiphyseal cartilages [7] and in the size and shape of the mandibular bone [8, 9], among other skeletal characteristics. For skeletal muscle, there is a difference in the number of fibers of masticatory muscle fibers (masseter, temporalis, and pterygoid) [10, 11], as well as a difference in fiber type composition and capillarity for other skeletal muscles [12]. At birth, females are 1% shorter than males, and this minor difference remains evident throughout childhood. During puberty, more substantial sexual dimorphic bone growth and developmental patterns arise [5]. Females reach puberty earlier and experience more rapid changes in bone and muscle than males, unlike men, who reach adulthood with more significant gains in muscle mass and bone mineral content (BMC), resulting in larger and stronger bones [2, 5, 13].

The dimorphic features of the musculoskeletal system have been mainly attributed to the action of sex steroid hormones [2, 6, 12, 14], which can influence maximum bone mass and architecture [7, 15] and musculature [2, 6, 12, 16, 17]. Until 1988, sex steroid hormones were thought to affect the skeleton only indirectly by regulating the secretion of the calciotropic hormones 1,25(OH)_2_ vitamin D_3_ and parathyroid hormone [18]. However, the advent of sensitive research techniques led to the discovery of sex steroid receptors in different bone cells, and revealed the direct actions of androgens and estrogens on bone [18]. For instance, androgens and estrogens directly affect the rate at which osteoclasts form, resorb bone, and die and directly affect bone-forming osteoblasts, often producing different effects in women and men [14, 19]. Furthermore, sex steroid hormones influence the survival of osteocytes and alter the coupling between bone formation and resorption, which is also known as the bone remodeling process [20]. Estrogen deficiency predisposes women to a greater risk of developing primary osteoporosis in menopause [5, 13, 21]. Similarly, the slower decline of testosterone with aging or the absence of the male hormone actions, as observed in men undergoing treatment for androgen-dependent prostate cancer, results in low bone mass in men [21]. Moreover, reduced or lack of sex steroids results in skeletal muscle atrophy in both sexes [12].

In this systematic review, we summarize the current literature on the ontogenic differences between the sexes/gender, with focus on the musculoskeletal system, and including the mandible and masticatory muscles (Fig. 1). We conducted an extensive electronic literature search in health-supported studies in human and preclinical rodent models and systematic/narrative reviews, not limiting our criteria to a specific range of years. We used MeSH terms and Boolean connectors with the keywords: Steroid Hormones, Bone Development, Muscle Development, Stomatognathic System, Sexual Characteristics/Dimorphisms, Mice, Male, Female, Mandible, Jaw, Transgender, Gender-Affirming Hormone Therapy, and Sex Chromosomes. The electronic databases we consulted were PubMed, Embase, Web of Science, and Scopus (Fig. 2).

**Figure 1.**
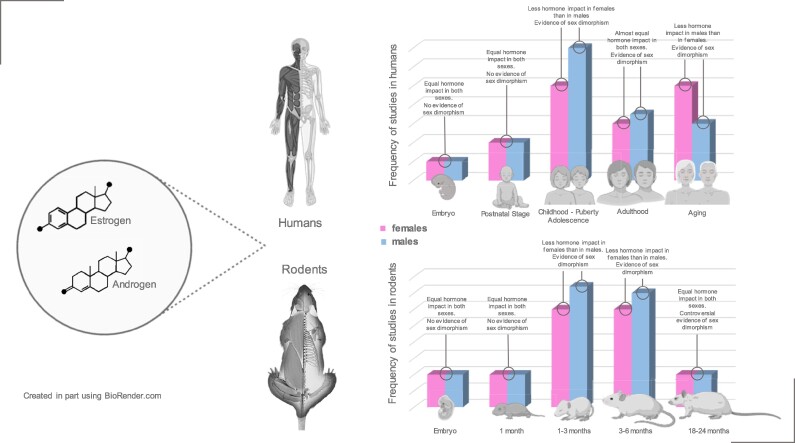
Sex hormones impact sexual dimorphism during musculoskeletal ontogeny. Schematic representation of the sexual dimorphism in the musculoskeletal system (including the jaw and masticatory muscles), and the relative contribution of male vs female hormones at each life stage. Scheme is based on reported studies investigating humans and rodents during development, growth, and aging. Created with BioRender.com.

**Figure 2.**
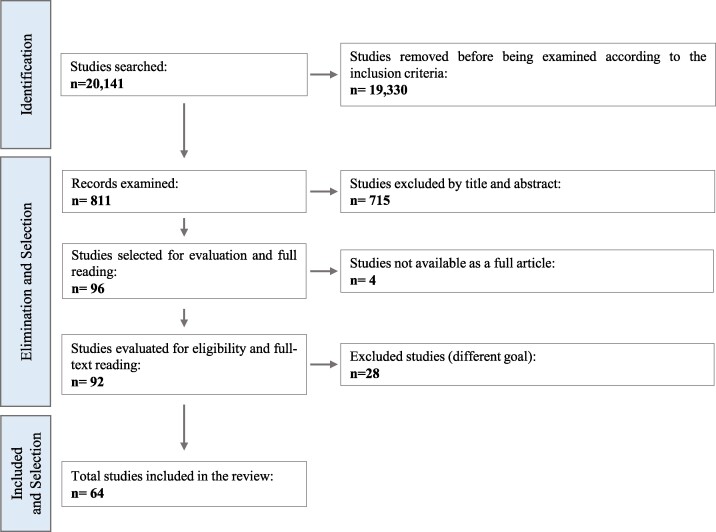
Flow chart depicting the search strategy for the current review. Bibliographic search and number of articles found, together with inclusion and exclusion criteria, as well as number of publications selected for the current review are indicated. Inclusion criteria include studies related to differences between sexes/genders during the ontogeny of the musculoskeletal system (including the mandible and masticatory muscles), health-supported studies in humans, preclinical rodent models, and systematic/narrative reviews. The bibliographic search was not limited by publication year for either human and rodent studies, and all publications dates that met the inclusion and exclusion criteria were considered. Created with BioRender.com.

This search yielded 20 941 articles written in English, from which we selected 64 that met all our search criteria.

##  

### Humans: Embryonic and Postnatal Stages

Bones are sculpted to achieve their unique shapes and sizes during intrauterine development and postnatal growth. The appendicular and axial bones are formed by a process known as endochondral ossification by which a cartilage template is replaced by mineralized bone tissue [22]. On the other hand, most bones of the skull (including the mandible, see below) and the scapula and clavicle are formed embryonically through intramembranous ossification. The first site of bone formation within the cartilage fundus is known as the primary ossification center, which develops before birth. In contrast, the bone patch that appears after birth is known as the secondary ossification center [22, 23]. There is increasing evidence that exposure to sex hormones during the fetal stage can have long-term effects on the bone phenotype, while the same sex hormone appears to affect male and female embryos in a similar manner [24], and, to our knowledge, there is no report of sexual dimorphism for skeletal muscle at this stage.

Facial development begins in the fourth week of embryonic age with the stomodeum, which becomes the mouth. In the sixth week, the mandibular processes increase in size and fuse in the midline to form the mandible, a product of the cartilaginous derivative of the first branchial arch called Meckel's cartilage [25, 26]. The oral ectoderm proliferates and forms the primary epithelial band, which becomes the alveolar processes of the maxilla and mandible, the vestibular lamina, and the dental lamina. The dental lamina forms the basis for the development of the dentition [25, 26]. Masticatory muscles are formed from the mesenchymal cells of the first branchial arch and exhibit a unique fiber type and size distribution compared with limbs and trunk muscles [8]. Despite sufficient evidence of the process described above, there are no reports of differences between sexes related to the growth and development of the mandible and masticatory muscles at the embryonic and postnatal stages in humans.

### Rodents: Embryonic and Postnatal Stages

To our knowledge, most studies investigating the contribution of gonadal sex on the ontogenic process focus on the mandible and masticatory muscles, but not other parts of the musculoskeletal system (Fig. 1). The murine mandible in the embryo appears as a thin plate of condensed mesenchymal cells, which proliferate and differentiate into osteogenic cells [27]. Each plate elongates and grows at its upper and lower edges, which fold medially and eventually surround Meckel's cartilage, similar to human mandibular development. Very little is known about how morphogenesis is controlled at this level because there are few reports on the molecular receptors expressed in the mandible and virtually none on the signal molecules expressed by Meckel's cartilage or nearby tissues [27]. Kinetic analysis has shown that cultured osteoprogenitor cells undergo 9 to 10 population doublings before the appearance of the first morphologically differentiated osteoblasts. Subsequently, the rodent mandible undergoes the necessary modifications to adapt to the growth of the masticatory muscles, which arise from the precordial mesoderm of the head or the neural crest, during growth and development [28]. As in humans, there are no reports investigating sexual dimorphism of the mandible during this early stage of rodent development. For the masticatory muscles, it has been reported that the proportion of different muscle fiber types differs markedly between males and females, although details on the fiber composition were not provided [11]. Differences arise for males during postnatal maturation whereas no changes were observed in females [11, 29]. Further, evidence indicates that testosterone is involved in the change in the proportion of muscle fibers in mice [11].

### Humans: From Childhood to Adolescence

Although BMC in the limbs and trunk is constant during growth [5], it has been reported that in both sexes the prepubertal growth rate is more significant in the appendicular skeleton than in the axial skeleton [24] (Fig. 1). Therefore, prepubertal children exhibit eunuchoid proportions (ie, long limbs in relation to the spine) [24]. Furthermore, size dimorphism is not very evident at this stage but becomes apparent at the onset of puberty. The 2 sexes take increasingly divergent paths during puberty: girls stop growing earlier than boys [5]. To reinforce this knowledge, a recent review reported that, although girls mature approximately 2 years earlier than boys (on average 11.8 vs 13.4 years), they are shorter on average [13] because boys continue pubertal growth for approximately 1 more year [5, 13]. This is the time when important sex differences in BMC emerge in all regions of the body [5], affecting leg length, overall bone size, and total bone mass [5, 13]. At this stage, sexual dimorphism in height is likely regulated by pubertal sex steroids [24]. Of note, most studies investigate the effect of androgens only in males and estrogens only in females. Thus, the adrenarche endocrine event represents an increase in the secretion of adrenal androgen hormones that accelerate height growth and bone maturation, among other phenomena in males [5]. In particular, puberty stimulates periosteal apposition, which is even more evident during adolescence [30] and not endocortical expansion resulting in increases in bone diameter, cortical thickness, and medullary diameter, particularly in the long bones of boys [5].

In females, estrogen preserves bone mass, suppresses bone turnover, and maintains balanced rates of bone formation and bone resorption while influencing the functional activity of bone cells and inducing epiphyseal closure [5]. Furthermore, estrogens inhibit periosteal apposition but stimulates endocortical bone formation so that in female puberty, there is an increase in cortical thickness with a decrease in medullary diameter and a slight increase in periosteal diameter [5]. Interestingly, testosterone also contributes to skeletal growth in females, but fails to exert its effects in the absence of estrogen [5].

Boys have a higher mineral mass in the appendicular skeleton, and girls have a significantly higher BMC and bone mineral density (BMD) in the spine and pelvis [5, 13, 31]. In contrast, in early postpubertal age, males show higher BMC and BMD than females, whereas females reach peak BMC and BMD earlier than males [31]. The contrasting effects of the sex hormones during puberty stage largely explain the greater skeletal size achieved by adult men [5, 24]. However, a recent mixed longitudinal study conducted in adolescents using high-resolution peripheral quantitative computed tomography showed no differences between sexes in cortical BMD [30]. Of note, this study compared boys and girls normalized to a common maturation landmark, the age at peak height velocity, defined as 11.6 years for girls and 13.5 years for boys. This suggests that the differences reported in other studies are related to different maturity stages rather than real sexual dimorphic effects.

Similar to bone, it has been reported that puberty is the stage at which sex differences in skeletal muscle are most evident [2, 5, 13]. These dimorphic characteristics are ascribed to high levels of testosterone and insulin-like growth factor-1, resulting in increased muscle mass and strength in boys, whereas estrogens tend to decrease muscle area in girls [6].

Sexual dimorphism in the human mandible has been reported to arise from the initial disparities in growth rate between boys and girls during childhood and puberty [8]. These differences already exist by age 9, and mainly affect size and not shape; mandibular size is usually bigger in boys than girls at this age. Although, both sexes show similar rates of size change between the ages of 9 and 12 years [8], boys show more remarkable changes in mandibular shape than girls between 12 and 14 years of age [32]. From that time until age 17, increases in mandibular size slows gradually in females, but not in males.

In the mandible, as in the long bones, accelerated postpubertal dimorphism results from changes in circulating hormone levels [33]. Specific genetic variants are known to influence circulating levels of sex hormones in humans, including variants in *SHBG*, the gene encoding sex hormone–binding globulin. Testosterone plays a fundamental role in these dimorphic characteristics of the masticatory muscles, in particular in the masseter muscle. Thus, androgens are required for the formation of fast-twitch muscle fibers, leading to reduced muscle fatigue, which is essential for chewing [16, 17].

### Rodents: Growing Animals

In studies with growing rodents [34], it has been shown that the tibia and radius increase in length faster in males than in females [7]. This sex difference in growth leads to higher maximum cortical bone mass in males. The growth processes involved in determining bone mass early in life includes endochondral ossification (bone length), secondary intramembranous ossification (cross-sectional area), and endocortical modeling (medullary area). At puberty [34], males develop greater body size and muscle mass, with more fast-twitch and fewer slow-twitch myofibers than females [2]. In addition, sexual dimorphic growth of skeletal muscle has been attributed to opposing actions of gonadal steroids, with androgens promoting growth and estrogens inhibiting it.

Researchers report differences in the shape and size of the murine mandibular bone, which is subject to dietary changes after weaning in wild and laboratory mice [35, 36]. The authors suggest that differences in shape appear greater in females, and that differences in size are predominately observed in males [35]. However, we were unable to identify any literature describing potential sexual dimorphism in the masticatory muscles of rodents at this stage (1-3 months, equivalent to adolescence in humans [34]).

### Humans: Adulthood and Aging

Differences in adult body size are best illustrated by height, which is, on average, 7% to 8% greater in males but also varies significantly between populations [5, 24] (Fig. 1). This difference is notable given that adult human height is a highly polygenic trait associated with approximately 697 genome-wide variants, including the estrogen receptor alpha gene *ESR1*. Skeletal weight varies between 4 kg in men and 2.8 kg in women at the beginning of adulthood. However, due to the height difference, although young men have higher BMC, their bone density is similar to that of young women [5]. Furthermore, adult men have a larger average rib cage volume and larger but relatively shorter and less curved ribs than adult women [37]. This could be due to an adaptation to potential pregnancy, as an expanded thoracic cavity can accommodate abdominal distension [37]. As adulthood progresses, sex steroid levels decrease due to an increase in sex hormone–binding proteins that reduce the concentration of the free form of the hormones [5]. This decrease could influence bones [5, 21], resulting in future sequelae such as the development of osteoporosis [5, 13, 21, 38].

It has been reported that adult men have a lower proportion of total BMC in the head than women [5] and that mandibular growth occurs during a longer period of time in men than in women [8]. Some studies have reported morphometric differences in adulthood, such as smaller mandibular sizes in women [39] or fewer variations in their mandibular morphometric measurements than in men [40]. The male mandible has more pronounced muscle insertions and exerts greater masticatory forces. These differences could be related to muscle fiber type content in men vs women, and possibly due to hormonal influences [10]; men have a higher prevalence of myosin heavy chain (MyHC) type IIb muscle fibers while women have primarily MyHC type IIa muscle fibers [11].

Aging also influences the skeletal phenotype, albeit in a different manner from the processes of growth and development [5]. The effect of pubertal growth is to increase bone size, while the effect of aging is endocortical bone resorption with a compensatory increase in periosteal apposition to enlarge the bone. Bone loss in aging includes trabecular thinning and loss of trabecular connectivity, and the rate of these processes differs between the sexes [5, 38]. The male vs female difference is influenced not only by the chronological aging process but also because women lose estrogens during menopause. However, bone loss begins long before the onset of menopause, and is accelerated after menopause due to the associated decrease in estrogen [5]. Menopause is associated with a 90% increase in bone resorption but only a 45% increase in bone formation, leading to an acceleration of bone loss in women, particularly on the endosteal surface [5, 21]. This period is followed by a slower phase of bone loss, which in part results from age-related abnormalities in calcium homeostasis in women, which also occur in elderly men [5, 21]. In fact, it is believed that the decrease in testosterone levels with aging influences bone loss in men in andropause [5]. Periosteal apposition is another factor affected by age, with greater bone formation occurring in men than in women. Overall, the distinct effects of aging on cancellous and cortical bone lead to different predispositions for fracture risk depending on the sex/gender [5]. Further, the loss of BMC is more significant in women than men at all skeletal sites, although the magnitude of the difference is greater in the spine and pelvis.

Both men and women suffer from skeletal muscle loss associated with a decrease in testosterone levels with aging. However, compared with women, men suffer a greater loss of muscle mass and strength throughout their lives [6]. In fact, men can experience transverse and longitudinal losses in muscle mass and strength that are twice those seen in women.

The aging process also impacts mandibular bone morphology and may influence sexual dimorphism in the mandible [3]. Studies confirm that these age-related changes are associated with sex hormones [9]. The decline in hormonal levels with advancing age is reflected in individuals of both sexes, but menopausal women undergo rapid mandibular bone replacement, whereas the decline is more gradual in men [41]. The earlier appearance of structural changes in women could be explained by decreases in estrogen levels during menopause, which causes more significant resorption of the facial bones, particularly the mandible, similar to what occurs with the long bones [41]. According to a 2021 study by Costa Mendes et al [3], in which age-related changes in mandibular shape and sexual dimorphism were evaluated in people aged between 40 and 79, mandibular sexual dimorphism in shape continued to be significant with aging. Conformational changes in the mandible were observed between 50 and 70 years of age and were different between men and women. Thus, women presented earlier and with more marked age-related changes in mandibular shape than men.

The dentition is the main determinant of mandibular morphology since tooth loss affects the height, length, and gonial angle of the mandibular body in both sexes [42]. Thus, age-related changes in men could be due to tooth loss. At the same time, in women, the effect of edentulism could be less striking due to the influence of hormonal factors [3]. Tooth loss leads to resorption of the alveolar bone ridge [42]. These observations suggest that mandibular senescence is a sexually dimorphic process, since conformational changes differ between male and female individuals. As for other muscles, age-related loss of masticatory muscle strength and function can lead to a decline in physical performance. Palinkas et al recorded a gradual decrease in resting masticatory muscle mass and thickness and maximum voluntary contraction in a study group of people aged over 60 [16].

### Rodents: Post-Skeletal Maturation and Aging

Male rodents reach a higher peak bone mass during growth than females, as is observed in humans [43]. Consistent with a higher number of osteoclasts in vivo in the femur of female mice, bone mass of female mice is lower than male mice [14].

In contrast, another study comparing the femurs of inbred mice showed that femurs of female mice had a larger cortical area than those of male mice (after adjusting for body size and bone size), unlike the evidence for humans [44]. Regarding sex-dependent effects on osteoclastogenesis, there has been controversy when evaluating osteoclast precursor cell cultures. Specifically, higher osteoclast differentiation and pronounced resorption have been observed in female mice than in cells obtained from male C57BL/6 mice [45], whereas it was found to be greater in males in male C57/129 mice [46], suggesting the cellular responses and/or the composition of the hematopoietic pool are influenced by genetic background. In rodents, it has also been reported that estrogens contribute to the adaptation of the female mouse skeleton for reproduction [7], and, in turn, testosterone reduces bone resorption and increases periosteal bone apposition in male mice [5, 20, 47]. Furthermore, testosterone has also been observed to rescue orchiectomy-induced bone loss in rodents, confirming the importance of androgen receptor signaling in regulating the male skeleton [48]. The essential role of sex hormones in mediating sex differences in rat tibial growth has been demonstrated by gonadectomy, which decreased bone growth in males but increased bone growth in females [49, 50]. Sex differences can be restored in gonadectomized animals by administering androgens to males to increase growth and estrogens to females to reduce it [7].

Regarding the masticatory system in adult rodents, studies have focused mostly on the muscles, and, in particular, on the development and behavior of the masseter muscle. This is appropriate because the masseter is the primary muscle that produces force to close the mandible in rodents, and it is also a sexually dimorphic muscle [11]. Daniels et al reported that specific isometric strength in the masseter muscle was not affected by sex in adult rodents [28]. However, contractive activity is faster in females than males, particularly in the ascent and relaxation movements. This feature is probably due to the more significant proportion of MyHC IIb fibers in the male deep anterior masseter muscle, which is the fiber type that produces the fastest rate of shortening [28]. Very few articles describe the sexual dimorphism of the masseter muscle of the adult mouse, so conclusions at this level cannot be definitive.

While rodents have been a useful tool to study sex differences in the musculoskeletal system, it should be considered that, unlike humans, rats and mice do not experience the abrupt loss of estrogen at menopause, nor do androgen levels appear to decrease with age in male mice [51]. However, when both rodents reach peak bone mass, gonadectomy of mice or rats older than 4 to 5 months of age closely replicates the loss of cancellous and cortical bone mass caused by estrogen or androgen deficiency in humans [24]. Because mice do not experience menopause, they are an invaluable model to analyze the contribution of sex steroid deficiency vs chronological aging on skeletal involution. Some studies have recently demonstrated that both male and female mice exhibit all the main characteristics of skeletal aging, including decreases in cortical and cancellous bone mass as well as the development of cortical porosity after 18 months of age [52]. These findings indicate that the effects of aging on the mammalian skeleton are independent of the effects of sex steroid deficiency [24].

On the other hand, it has been reported that in female rodents, the content of mineralized mandibular alveolar bone is significantly reduced compared with old male rodents, which the authors proposed is related to hormonal influence [53], as is observed in humans [9]. In contrast to the above, insufficient evidence was found to explain the mechanism for aging and the influence of sex steroid hormones on the masticatory muscles in rodents.

### The Impact of Cross-sex or Gender-affirming Hormone Therapy on the Musculoskeletal System

Cross-sex hormone therapy (CSHT) also known as gender-affirming hormone therapy suppresses gonadal hormones and secondary sexual characteristics of the individual's sex at birth and induces bodily characteristics consistent with their gender identity [54, 55] (Fig. 1). Regarding the effects of CSHT on bone mass, 2 systematic reviews have been published, evaluating the impact of CSHT on bone mass [56, 57]. However, no definitive conclusions have been reached. Some authors have reported no significant difference in BMD in transgender men receiving CSHT whereas lumbar spine BMD is increased in transgender women receiving CSHT. In contrast, other studies report that before CSHT, transgender women tend to have lower BMD and a higher prevalence of osteopenia than cisgender men. Further, implementation of CSHT restores or at least improves BMD in both transgender boys and girls. These authors also emphasize that short-term administration of CSHT does not appear to have a negative effect on BMD in adult transgender women and men and that gonadectomy is not a risk factor if CSHT is taken correctly. These inconclusive results could be influenced by the type and route of administration of estrogens and testosterone used for CSHT, type of treatment regimens, the interpretation of body mass index values, as well as the effect of gonadotropin-releasing hormone (GnRH) agonists, gonadectomy and antiandrogens, and even the influence of ethnic origin and lifestyle habits on BMD.

Regarding muscles, after 12 months of CSHT, an increase in muscle mass and strength has been observed in transgender men while muscle mass is slightly decreased or unchanged in transgender women [58]. In a recent review where the effect of CSHT on the muscles was measured 2 years after CSHT, the mass and strength muscle mass were found to be increased in transgender men. In contrast, an increase in fat mass of approximately 30% and a decrease in skeletal muscle mass of approximately 5% was observed in transgender women treated with CSHT for 12 months, and continued to decrease steadily beyond 3 years [59].

In humans, male to female transition is achieved in adolescents in whom puberty is first arrested by the use of GnRH analogues with subsequent treatment with estradiol. In rodents, more complex studies of the effects of experimental male to female transition are possible. For instance, orchiectomized mice, which exhibit decreased maximum bone mass accumulation and a decrease in the maximum force that the bone could withstand before fracture, were given estradiol to simulate male to female transition [60]. This resulted in increased cortical thickness in the midshaft. Estradiol treatment also increased newly formed trabecular bone, mineralized surface area/bone surface and bone formation rate, all of which are related to the anabolic action of estradiol on osteoblast proliferation. Likewise, in another study where adult male mice were treated with a sustained high dose of estradiol, endocortical bone deposition was stimulated in the femoral midshaft, increasing cortical thickness and bone area. Treatment with high doses of estradiol was also anabolic for trabecular bone, notably increasing the volume, quantity, and thickness of trabecular bone in the distal metaphysis, which was accompanied by an increase in histomorphometric markers of bone remodeling, surface mineralized/bone surface, bone formation rate, and number of osteoclasts. These findings in rodents support the need to optimize the doses of estradiol administration in human transgender women [61].

### Not Just Sex Hormones—Role of Sex Chromosomes on the Musculoskeletal System

Sexual dimorphism has been ascribed to the relative levels of gonadal hormones in males (androgens) vs females (estrogens) [24, 62]. However, there has been increased interest in determining the potential role of the sex chromosomes in the different tissues and organs. To tease out the potential contribution of gonadal (presence of ovaries or testis) vs chromosome sex (presence of XX or XY chromosomes) to the differential male/female effects, a mouse model named the four-core genotypes (FCG) was developed [63]. In these mice, the testis-determining gene *Sry* was deleted from the Y chromosome and/or expressed as a transgene. Using this strategy, mice of 2 sexes are generated, depending on the gonad present: male XXM (*Sry* transgenic with testes) or XY^−sry^M (*Sry*-deficient Y chromosome and Sry transgenic with testes), and female XXF (wild type with ovaries) or XY^−sry^F (*Sry*-deficient Y chromosome with ovaries). Studies with these mice uncovered chromosome sex effects on the brain, lifespan, and adiposity, unrelated to the sex hormone present [63-65]. Our laboratory has used these mice to understand the consequences of these genetic manipulations on the musculoskeletal system in young (2-month-old) and adult (4-month-old) mice [66]. We found that while gonadal sex is the main contributor to the differences in bone and skeletal muscle mass at 2 months of age, the sex chromosome complement (ie, XX vs XY) impacts fat mass at this age. By 4 months of age, the contribution of the sex chromosomes is more evident, with differences observed in bone mass and strength, bone cell number/activity, body weight and composition, and skeletal muscle mass, depending on the presence of XX vs XY chromosomes within each gonadal sex. It should be noted, however, that it has been shown that a 3.2 Mb translocation of a segment of near-PAR X chromosome to the Y^−sry^ chromosome. Therefore, a potential contribution of the duplicated genes on the phenotypes should be considered when interpreting the results the from results from this mouse model.

## Conclusions

The evidence presented in this review summarizes the role of sex/gender on the musculoskeletal ontogenic process. Current literature is primarily focused on childhood/puberty to adulthood where it has been shown in both humans and rodents that males exhibit more pronounced musculoskeletal changes than females. Only a handful of studies have investigated the effects of aging stage in rodents, and no differences between the sexes were identified. The evidence for older humans was more numerous and concluded that women experience greater changes in bone and skeletal muscle integrity and function than men, likely due to menopause. More longitudinal side by side studies are needed to better understand the mechanisms underlying differences between men and women in bone and muscle ontogeny and involution with age. Furthermore, it is expected that future research will describe the sex-specific ontogeny of the stomatognathic system (ie, the jaw and accompanying skeletal muscles) together with that of the musculoskeletal system (Fig. 1).

## Data Availability

No original data was included in this manuscript.

## Abbreviations

BMC: bone mineral content
BMD: bone mineral density
CSHT: cross-sex hormone therapy
GnRH: gonadotropin-releasing hormone
FCG: four-core genotypes
MyHC: myosin heavy chain

## References

[1] Burr DB . Bone mophology and organization. In: Burr DB, Allen MR, eds. Basic and Applied Bone Biology. Elsevier; 2019:3‐26.

[2] Paul RG, Hennebry AS, Elston MS, et al Regulation of murine skeletal muscle growth by STAT5B is age- and sex-specific. Skelet Muscle. 2019;9(1):19. 31230596 10.1186/s13395-019-0204-3 PMC6589877

[3] Costa Mendes L, Delrieu J, Gillet C, et al Sexual dimorphism of the mandibular conformational changes in aging human adults: a multislice computed tomographic study by geometric morphometrics. PLoS One. 2021;16(6):e0253564. 34157047 10.1371/journal.pone.0253564 PMC8219137

[4] Bragdon B, Burns R, Baker AH, et al Intrinsic sex-linked variations in osteogenic and adipogenic differentiation potential of bone marrow multipotent stromal cells. J Cell Physiol. 2015;230(2):296‐307. 24962433 10.1002/jcp.24705 PMC4317374

[5] Wells JC . Sexual dimorphism of body composition. Best Pract Res Clin Endocrinol Metab. 2007;21(3):415‐430. 17875489 10.1016/j.beem.2007.04.007

[6] Lang TF . The bone-muscle relationship in men and women. J Osteoporos. 2011;2011:1‐4. 10.4061/2011/702735 PMC3189615 22007336

[7] Turner RT . Mice, estrogen, and postmenopausal osteoporosis. J Bone Miner Res. 1999;14(2):187‐191. 9933471 10.1359/jbmr.1999.14.2.187

[8] Fan Y, Penington A, Kilpatrick N, et al Quantification of mandibular sexual dimorphism during adolescence. J Anat. 2019;234(5):709‐717. 30834524 10.1111/joa.12949 PMC6481415

[9] Toneva DH, Nikolova SY, Fileva NF, et al Size and shape of human mandible: sex differences and influence of age on sex estimation accuracy. Leg Med (Tokyo). 2023;65:102322. 37722156 10.1016/j.legalmed.2023.102322

[10] van Selms MKA, Wang K, Lobbezoo F, Svensson P, Arendt-Nielsen L, Naeije M. Effects of masticatory muscle fatigue without and with experimental pain on jaw-stretch reflexes in healthy men and women. Clin Neurophysiol. 2005;116(6):1415‐1423. 15978504 10.1016/j.clinph.2005.02.017

[11] Eason JM, Schwartz GA, Pavlath GK, English AW. Sexually dimorphic expression of myosin heavy chains in the adult mouse masseter. J Appl Physiol (1985). 2000;89(1):251‐258. 10904059 10.1152/jappl.2000.89.1.251

[12] Della Peruta C, Lozanoska-Ochser B, Renzini A, et al Sex differences in inflammation and muscle wasting in aging and disease. Int J Mol Sci. 2023;24(5):4651. 36902081 10.3390/ijms24054651 PMC10003083

[13] Ortona E, Pagano MT, Capossela L, Malorni W. The role of sex differences in bone health and healing. Biology (Basel). 2023;12(7):993. 37508423 10.3390/biology12070993 PMC10376157

[14] Mun SH, Jastrzebski S, Kalinowski J, et al Sexual dimorphism in differentiating osteoclast precursors demonstrates enhanced inflammatory pathway activation in female cells. J Bone Miner Res. 2020;36(6):1104‐1116. 10.1002/jbmr.4270 PMC11140852 33567098

[15] Laurent M, Antonio L, Sinnesael M, et al Androgens and estrogens in skeletal sexual dimorphism. Asian J Androl. 2014;16(2):213‐222. 24385015 10.4103/1008-682X.122356 PMC3955330

[16] Palinkas M, Nassar MSP, Cecílio FA, et al Age and gender influence on maximal bite force and masticatory muscles thickness. Arch Oral Biol. 2010;55(10):797‐802. 20667521 10.1016/j.archoralbio.2010.06.016

[17] Galhardo APM, Mukai MK, Mori M, et al Influence of age and gender on sex steroid receptors in rat masticatory muscles. Sci Rep. 2019;9(1):18403. 31804540 10.1038/s41598-019-54774-y PMC6895217

[18] Chang CS, Kokontis J, Liao ST. Molecular cloning of human and rat complementary DNA encoding androgen receptors. Science. 1988;240(4850):324‐326. 3353726 10.1126/science.3353726

[19] Lorenzo J . Sexual dimorphism in osteoclasts. Cells. 2020;9(9):2086. 32932615 10.3390/cells9092086 PMC7564933

[20] Sandor LF, Ragacs R, Gyori DS. Local effects of steroid hormones within the bone microenvironment. Int J Mol Sci. 2023;24(24):17482. 38139309 10.3390/ijms242417482 PMC10744126

[21] Zhang Y-Y, Xie N, Sun X-D, et al Insights and implications of sexual dimorphism in osteoporosis. Bone Res. 2024;12(1):8. 38368422 10.1038/s41413-023-00306-4 PMC10874461

[22] Allen MR, Burr DB. Bone growth, modeling, and remodeling. In: Allen MR, Burr DB, eds. Basic and Applied Bone Biology. Elsevier; 2019:85‐100.

[23] Al-Khater KM, Hegazi TM, Al-Thani HF, et al Time of appearance of ossification centers in carpal bones. Saudi Med J. 2020;41(9):938‐946. 32893275 10.15537/smj.2020.9.25348 PMC7557557

[24] Almeida M, Laurent MR, Dubois V, et al Estrogens and androgens in skeletal physiology and pathophysiology. Physiol Rev. 2017;97(1):135‐187. 27807202 10.1152/physrev.00033.2015 PMC5539371

[25] Mello-Gentil T, Souza-Mello V. Contributions of anatomy to forensic sex estimation: focus on head and neck bones. Forensic Sci Res. 2022;7(1):11‐23. 35341126 10.1080/20961790.2021.1889136 PMC8942509

[26] Zohrabian VM, Poon CS, Abrahams JJ. Embryology and anatomy of the jaw and dentition. Semin Ultrasound CT MR. 2015;36(5):397‐406. 26589693 10.1053/j.sult.2015.08.002

[27] Ramaesh T, Bard JBL. The growth and morphogenesis of the early mouse mandible: a quantitative analysis. J Anat. 2003;203(2):213‐222. 12924821 10.1046/j.1469-7580.2003.00210.x PMC1571157

[28] Daniels DW, Tian Z, Barton ER. Sexual dimorphism of murine masticatory muscle function. Arch Oral Biol. 2008;53(2):187‐192. 18028868 10.1016/j.archoralbio.2007.09.006 PMC2262833

[29] Vora SR, Camci ED, Cox TC. Postnatal ontogeny of the cranial base and craniofacial Skeleton in male C57BL/6J mice: a reference standard for quantitative analysis. Front Physiol. 2016;6:417. 26793119 10.3389/fphys.2015.00417 PMC4709510

[30] Gabel L, Macdonald HM, McKay HA. Sex differences and growth-related adaptations in bone microarchitecture, geometry, density, and strength from childhood to early adulthood: a mixed longitudinal HR-pQCT study. J Bone Miner Res. 2017;32(2):250‐263. 27556581 10.1002/jbmr.2982 PMC5233447

[31] Nguyen TV, Maynard LM, Towne B, et al Sex differences in bone mass acquisition during growth: the Fels Longitudinal study. J Clin Densitom. 2001;4(2):147‐157. 11477308 10.1385/jcd:4:2:147

[32] Katsadouris A, Halazonetis DJ. Geometric morphometric analysis of craniofacial growth between the ages of 12 and 14 in normal humans. Eur J Orthod. 2016;39(4):386‐394. 10.1093/ejo/cjw070 27940444

[33] Roosenboom J, Indencleef K, Lee MK, et al SNPs associated with testosterone levels influence human facial morphology. Front Genet. 2018;9:497. 30405702 10.3389/fgene.2018.00497 PMC6206510

[34] JAX® Mice, C.R.S . Aged C57BL/6J mice for research studies: Considerations, applications and best practices. 2017.

[35] Martínez-Vargas J, Muñoz-Muñoz F, Martinez-Maza C, Molinero A, Ventura J. Postnatal mandible growth in wild and laboratory mice: differences revealed from bone remodeling patterns and geometric morphometrics. J Morphol. 2017;278(8):1058‐1074. 28503758 10.1002/jmor.20694

[36] Renaud S, Auffray J-C, De La Porte S. Epigenetic effects on the mouse mandible: common features and discrepancies in remodeling due to muscular dystrophy and response to food consistency. BMC Evol Biol. 2010;10(1):28. 20105331 10.1186/1471-2148-10-28 PMC2827398

[37] Beresheim AC, Pfeiffer S, Grynpas M. Ontogenetic changes to bone microstructure in an archaeologically derived sample of human ribs. J Anat. 2020;236(3):448‐462. 31729033 10.1111/joa.13116 PMC7018627

[38] Choi KH, Lee JH, Lee DG. Sex-related differences in bone metabolism in osteoporosis observational study. Medicine (Baltimore). 2021;100(21):e26153. 34032772 10.1097/MD.0000000000026153 PMC8154389

[39] Tomomi H, Tsukasa S, Kenji S, Tomohiro O. Radiologic measurements of the mandible: a comparison between CT-reformatted and conventional tomographic images. Clin Oral Implants Res. 2004;15(2):226‐232. 15008935 10.1111/j.1600-0501.2004.00991.x

[40] Oettlé AC, Pretorius E, Steyn M. Geometric morphometric analysis of the use of mandibular gonial eversion in sex determination. Homo. 2009;60(1):29‐43. 18996521 10.1016/j.jchb.2007.01.003

[41] Windhager S, Mitteroecker P, Rupić I, Lauc T, Polašek O, Schaefer K. Facial aging trajectories: a common shape pattern in male and female faces is disrupted after menopause. Am J Phys Anthropol. 2019;169(4):678‐688. 31189026 10.1002/ajpa.23878 PMC6771603

[42] Kuć J, Sierpińska T, Gołębiewska M. The relationship between facial morphology and the structure of the alveolar part of the mandible in edentulous complete denture wearers. A preliminary study. Acta Odontol Scand. 2015;73(1):57‐66. 25183253 10.3109/00016357.2014.950181

[43] Zanotti S, Kalajzic I, Aguila HL, Canalis E. Sex and genetic factors determine osteoblastic differentiation potential of murine bone marrow stromal cells. PLoS One. 2014;9(1):e86757. 24489784 10.1371/journal.pone.0086757 PMC3904935

[44] Jepsen KJ, Bigelow EMR, Schlecht SH. Women build long bones with less cortical mass relative to body size and bone size compared with men. Clin Orthop Relat Res. 2015;473(8):2530‐2539. 25690167 10.1007/s11999-015-4184-2 PMC4488191

[45] Paglia DN, Yang X, Kalinowski J, Jastrzebski S, Drissi H, Lorenzo J. Runx1 regulates myeloid precursor differentiation into osteoclasts without affecting differentiation into antigen presenting or phagocytic cells in both males and females. Endocrinology. 2016;157(8):3058‐3069. 27267711 10.1210/en.2015-2037 PMC4967120

[46] Valerio MS, Basilakos DS, Kirkpatrick JE, et al Sex-based differential regulation of bacterial-induced bone resorption. J Periodontal Res. 2017;52(3):377‐387. 27509894 10.1111/jre.12401 PMC5303566

[47] Maclean HE, Moore AJ, Sastra SA, et al DNA-binding-dependent androgen receptor signaling contributes to gender differences and has physiological actions in males and females. J Endocrinol. 2010;206(1):93‐103. 20395380 10.1677/JOE-10-0026

[48] Venken K, De Gendt K, Boonen S, et al Relative impact of androgen and estrogen receptor activation in the effects of androgens on trabecular and cortical bone in growing male mice: a study in the androgen receptor knockout mouse model. J Bone Miner Res. 2006;21(4):576‐585. 16598378 10.1359/jbmr.060103

[49] Wiren KM, Zhang X-W, Toombs AR, et al Targeted overexpression of androgen receptor in osteoblasts: unexpected Complex bone phenotype in growing animals. Endocrinology. 2004;145(7):3507‐3522. 15131013 10.1210/en.2003-1016

[50] Kousteni S, Chen J-R, Bellido T, et al Reversal of bone loss in mice by nongenotropic signaling of sex steroids. Science. 2002;298(5594):843‐846. 12399595 10.1126/science.1074935

[51] Almeida M, Han L, Martin-Millan M, et al Skeletal involution by age-associated oxidative stress and its acceleration by loss of sex steroids. J Biol Chem. 2007;282(37):27285‐27297. 17623659 10.1074/jbc.M702810200 PMC3119455

[52] Ucer S, Iyer S, Kim H-N, et al The effects of aging and sex steroid deficiency on the murine Skeleton are independent and mechanistically distinct. J Bone Miner Res. 2017;32(3):560‐574. 27714847 10.1002/jbmr.3014 PMC5340621

[53] Biguetti CC, Lakkasetter Chandrashekar B, Simionato GB, et al Influence of age and gender on alveolar bone healing post tooth extraction in 129 Sv mice: a microtomographic, histological, and biochemical characterization. Clin Oral Investig. 2023;27(8):4605‐4616. 10.1007/s00784-023-05087-y 37261497

[54] Fighera TM, Ziegelmann PK, Rasia da Silva T, Spritzer PM. Bone mass effects of cross-sex hormone therapy in transgender people: updated systematic review and meta-analysis. J Endocr Soc. 2019;3(5):943‐964. 31020058 10.1210/js.2018-00413 PMC6469959

[55] Singh-Ospina N, Maraka S, Rodriguez-Gutierrez R, et al Effect of sex steroids on the bone health of transgender individuals: a systematic review and meta-analysis. J Clin Endocrinol Metab. 2017;102(11):3904‐3913. 28945851 10.1210/jc.2017-01642

[56] Giacomelli G, Meriggiola MC. Bone health in transgender people: a narrative review. Ther Adv Endocrinol Metab. 2022;13:204201882210993. 10.1177/20420188221099346 PMC9150228 35651988

[57] Rothman MS, Iwamoto SJ. Bone health in the transgender population. Clin Rev Bone Miner Metab. 2019;17(2):77‐85. 31452648 10.1007/s12018-019-09261-3 PMC6709704

[58] Wiik A, Lundberg TR, Rullman E, et al Muscle strength, size, and composition following 12 months of gender-affirming treatment in transgender individuals. J Clin Endocrinol Metab. 2020;105(3):e805‐e813. 10.1210/clinem/dgz247 31794605

[59] Cheung AS, Zwickl S, Miller K, et al The impact of gender-affirming hormone therapy on physical performance. J Clin Endocrinol Metab. 2024;109(2):e455‐e465. 37437247 10.1210/clinem/dgad414 PMC10795902

[60] Nie T, Venkatesh VS, Golub S, et al Estradiol increases cortical and trabecular bone accrual and bone strength in an adolescent male-to-female mouse model of gender-affirming hormone therapy. Bone Res. 2024;12(1):1. 38212599 10.1038/s41413-023-00308-2 PMC10784310

[61] Venkatesh VS, Nie T, Golub S, et al High circulating concentrations of estradiol are anabolic for bone mass and strength in an adult male to female transgender mouse model. Bone. 2024;186:117143. 38866125 10.1016/j.bone.2024.117143

[62] Rigby N, Kulathinal RJ. Genetic architecture of sexual dimorphism in humans. J Cell Physiol. 2015;230(10):2304‐2310. 25740260 10.1002/jcp.24979

[63] Arnold AP, Chen X. What does the “four core genotypes” mouse model tell US about sex differences in the brain and other tissues? Front Neuroendocrinol. 2009;30(1):1‐9. 19028515 10.1016/j.yfrne.2008.11.001 PMC3282561

[64] Davis EJ, Lobach I, Dubal DB. Female XX sex chromosomes increase survival and extend lifespan in aging mice. Aging Cell. 2019;18(1):e12871. 30560587 10.1111/acel.12871 PMC6351820

[65] Chen X, McClusky R, Chen J, et al The number of x chromosomes causes sex differences in adiposity in mice. PLoS Genet. 2012;8(5):e1002709. 22589744 10.1371/journal.pgen.1002709 PMC3349739

[66] Ramirez G, Okpara C, Arnett M, et al Independent contribution of gonads and sex chromosomes to sex differences in bone mass and strength in the Four-Core Genotypes mouse model. J Bone Miner Res. 2024. Doi: 10.1093/jbmr/zjae147 PMC11523188 39255371

